# Predictive factors for spontaneous dislodgement of percutaneous nephrostomies for malignant ureteral obstruction

**DOI:** 10.1007/s00261-025-04855-6

**Published:** 2025-02-26

**Authors:** Ayşe Rüksan Ütebey, Halil Serdar Aslan, Muhammet Arslan, Kadir Han Alver, Hakkı Peker, Muhammed Tekinhatun, Ahmet Baki Yağcı, Nuran Sabir

**Affiliations:** 1 Pamukkale University, Denizli, Turkey; 2 Dicle University, Diyarbakır, Turkey

**Keywords:** Spontaneous percutaneous nephrostomy (PCN) catheter dislodgements, Malignant üreteral obstruction (MUO), Body fat-to-muscle composition and renal tissue parameters, Eastern cooperative oncology group (ECOG) performance status

## Abstract

**Purpose:**

To investigate the etiology of spontaneous percutaneous nephrostomy (PCN) catheter dislodgements and evaluate factors potentially associated with these dislodgements, including muscle-to-fat composition and tissue characteristics of catheter traces.

**Materials and methods:**

Data from 92 patients (63 males, 29 females; mean age 63.9 ± 11.4 years, range 28–88) undergoing 151 PCN catheter replacements between January 2016 and June 2021 were analyzed. Patients were divided into Group 1 (prophylactic replacements every 3 months, *n* = 41) and Group 2 (at least one spontaneous dislodgement, *n* = 51). Associations were evaluated for factors including intraabdominal visceral adipose tissue index (IAVATI), subcutaneous adipose tissue index (SATI), and abdominal perimeter. Other variables assessed were Eastern Cooperative Oncology Group (ECOG) performance status scores, psoas muscle index (PMI), renal size, renal parenchymal thickness, renal cortex-to-skin distance, posterolateral abdominal wall muscle thickness, and PCN replacement frequency.

**Results:**

No significant differences were identified between Group 1 and Group 2 in IAVATI, SATI, or abdominal perimeter values (*p* = 0.210–0.412). A significant difference in ECOG performance status scores (*p* = 0.0001), PMI (*p* = 0.04) and lower renal size, renal parenchymal thickness, renal cortex-to-skin distance, and posterolateral abdominal muscle thickness (*p* = 0.0001–0.039) were observed in Group 2. PCN replacements were significantly more frequent in Group 2 (*p* = 0.0001). Multivariate regression identified renal parenchymal thickness and abdominal wall muscle thickness as significant independent predictors (*p* = 0.0001, *p* = 0.02). ROC analysis yielded an AUC of 0.843 (95% CI: 0.769–0.917) for renal parenchymal thickness and 0.694 (95% CI: 0.610–0.778) for abdominal wall muscle thickness. Sensitivity and specificity rates were 73.1% and 96.4% for a 16 mm cutoff in renal parenchymal thickness, and 50.7% and 79.8% for an 8 mm cutoff in abdominal wall muscle thickness.

**Conclusion:**

A significant association was identified between spontaneous PCN catheter dislodgement and both the psoas muscle index and ECOG performance status scores, while no notable relationship was observed with abdominal visceral or subcutaneous fat tissue volumes or abdominal perimeter. The risk of dislodgement was found to increase with reduced renal parenchymal and abdominal wall muscle thickness, as well as with more frequent nephrostomy replacements, suggesting these parameters may serve as useful markers for identifying patients at higher risk.

**Graphical abstract:**

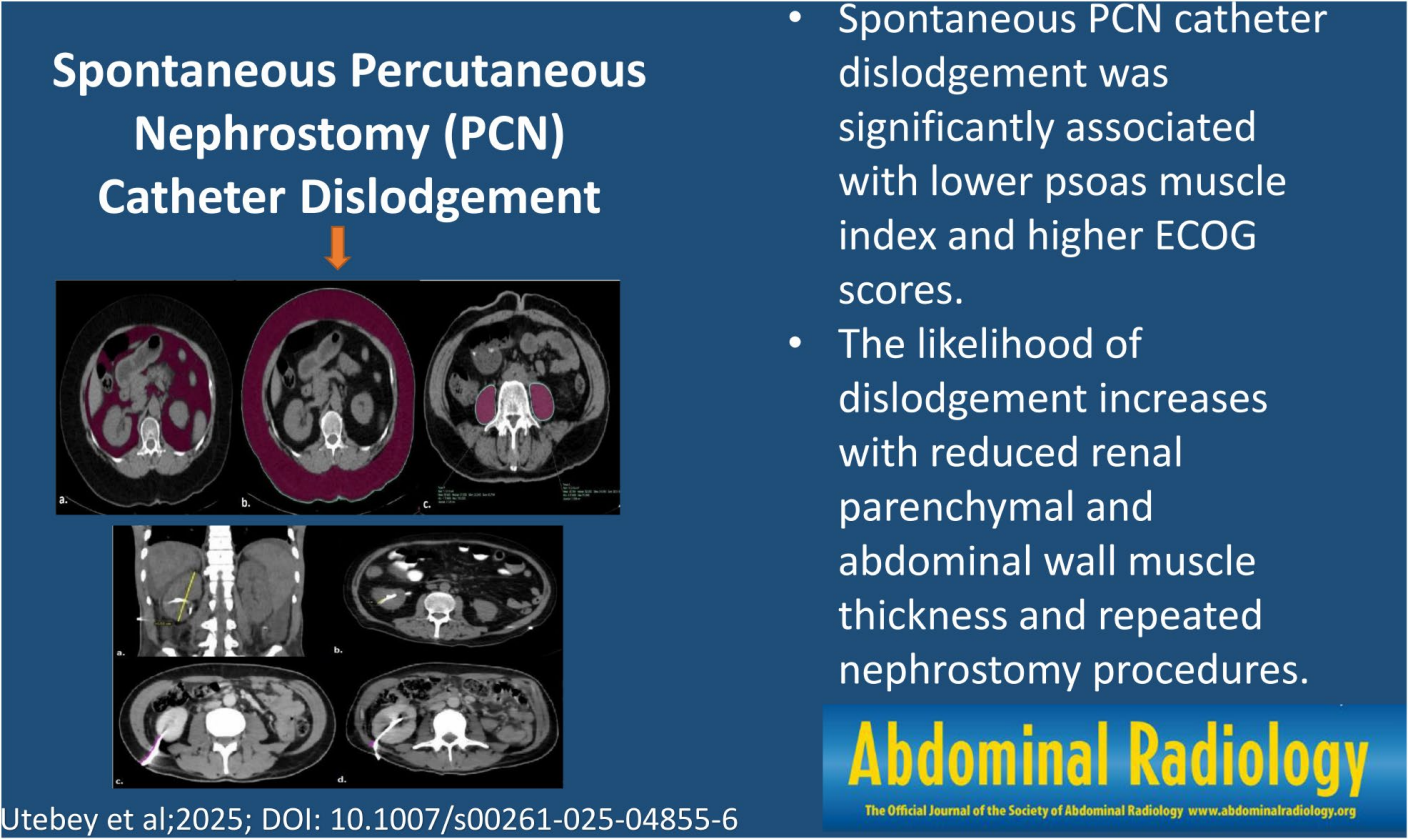

## Introduction

Percutaneous nephrostomy (PCN) is a minimally invasive procedure in which a flexible catheter is placed into the renal collecting system through the skin under ultrasound and/or fluoroscopic imaging in order to maintain external drainage of urine [1]. In the majority of cases, PCN is usually performed to relieve urinary obstruction due to benign causes (stones, strictures, pyonephrosis etc.) and malignancies, obtain urinary diversion for treatment of urinary fistulas and leaks and provide access to the collecting system for subsequent interventional procedures [2]. Collecting system obstruction due to malignant diseases is the main indication for PCN in more than 50% of patients in some series and a considerable number of those patients require long term follow-up with nephrostomy catheters [3]. Those patients requiring persistent nephrostomy due to malignant ureteral obstruction become more prone to catheter-related complications like catheter blockage and dislodgement. Pigtail, Malecot and balloon retention catheters are the most available and commonly used ones which have pull-string tethered locking systems, wings and inflatable balloons respectively for fixing tubes to the collecting system and promoting catheter retention [4]. Following the placement of nephrostomy catheter into the pelvis, all catheters are secured to the skin at the puncture site with multiple stitches and knots to avoid dislodgement. Additionally all patients and companions are instructed clearly and in detail about catheter care and what needs to be done to prevent catheter dislodgement [5]. Despite all the precautions outlined, spontaneous nephrostomy catheter dislodgements continue to be among the most significant catheter-related complications, with reported rates in the literature ranging from 1 to 73.5% [1, 6–12].

The literature contains limited data, with only a few studies investigating the predisposing factors for PCN catheter dislodgement [7, 9, 13]. To the best of our knowledge, some crucial factors and key determinants potentially related to PCN catheter dislodgement, such as sarcopenia, tissue characteristics and thickness (including renal size, renal parenchymal thickness, renal cortex-to-skin distance, and posterolateral abdominal wall muscle thickness), as well as body fat-to-muscle composition parameters (such as abdominal perimeter, and intraabdominal and subcutaneous adipose tissue area), have not been adequately addressed or discussed in the literature.

Thus, this study aimed to investigate the aforementioned factors, as well as other factors possibly associated with spontaneous PCN dislodgement, which is one of the most important long-term complications commonly seen in patients requiring recurrent nephrostomy interventions due to malignant ureteral obstructions.

## Materials and methods

### Patient selection

This retrospective, single-center study was initiated following approval from the local ethics committee (Approval number: 13/13072021). Before undergoing PCN procedures, all patients provided informed consent after being thoroughly informed about the procedure and its potential risks. Data were collected from electronic medical records and the Picture Archiving and Communication System (PACS), including patients’ clinical history, Eastern Cooperative Oncology Group (ECOG) performance status scores, body mass index values, demographic details, the number of PCN dislodgement events, and the median catheter dislodgement time (days). Between January 2016 and June 2021, a total of 332 patients who underwent PCN catheter replacement procedures at our clinic were evaluated. Among these, 92 patients (63 males, 29 females; mean age 63.9 ± 11.4 years, range 28–88) meeting the following criteria were included in the study. These patients underwent a total of 151 PCN catheter replacements.

### Inclusion criteria

1. **Patients without PCN Catheter Dislodgement (Group 1)**: This group included patients with long-term PCN catheters placed due to malignancy, whose catheters were replaced prophylactically at three-month intervals without any history of catheter dislodgement.
2. **Patients with PCN Catheter Dislodgement (Group 2)**: This group also consisted of patients with long-term PCN catheters placed due to malignancy. Patients included were those with at least one documented episode of inadvertent and spontaneous catheter dislodgement in their medical history or those whose PCN catheters were replaced due to dislodgement.

### Exclusion criteria

The following patients were excluded from the study:

- Those who underwent PCN replacement for benign causes, including benign prostatic hyperplasia, urinary tract stone disease, ureteral leak or fistula, and ureteral stricture.
- Patients without a computed tomography (CT) scan within two months before or after the nephrostomy procedure.
- Patients having CT examinations with significant imaging artifacts (e.g., motion or beam hardening) causing density changes in soft tissues.
- Cases in which the nephrostomy catheter dislodged due to accidental or traumatic causes.
- Patients with the need for recurrent PCN eliminated by the insertion of a double J stent via anterograde approach.
- Cases with renal rotation or axis anomalies.
- Bedridden patients (Patients with an ECOG performance status score of 4).
- Patients with scoliosis.
- Cases with infections, abscesses, or hematomas along the nephrostomy tract.

### PCN catheter replacement Procedure

All PCN catheter placements included in the study were performed by two radiologists with 15 and 8 years of experience in interventional radiology. All patients underwent a complete blood count and coagulation studies on the day of the procedure. Acceptable pre-procedure criteria included platelets > 50,000/µL and INR < 1.5. Anticoagulant therapy was assessed, and according to SIR guidelines, coumadin and antiplatelet drugs such as plavix and aspirin were stopped five days before. Bridging therapy with low molecular weight heparin was provided for patients with recently placed stents, mechanical heart valves, or peripheral artery disease. Unfractionated heparin was discontinued 2–3 h before the procedure, and fractionated heparin, such as enoxaparin, was withheld for 24 h prior to the procedure [14, 15].

**Fig. 1:**
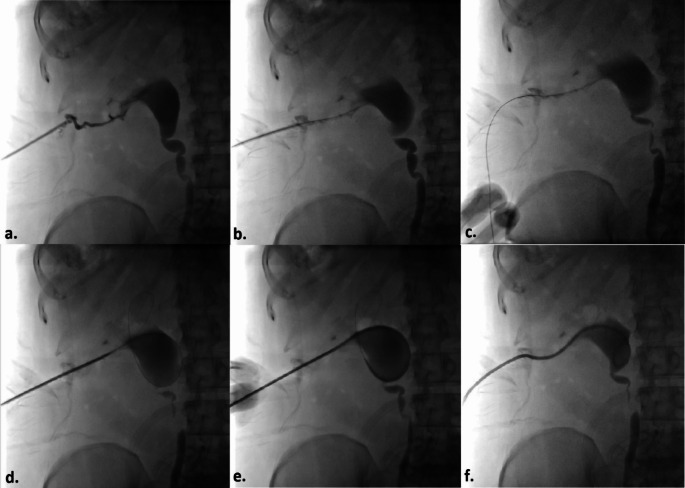
Percutaneous Nephtrostomy Via an Immaturated Tract **(a)** With the patient in the prone position, an 8-French (8 F) dilator is inserted at the skin entry point, and the existing immature tract from the skin to the collecting system is opacified by administering contrast material under fluoroscopic guidance. **(b)** A 0.035-inch hydrophilic guidewire is advanced through the 8 F dilator towards the renal pelvis. **(c)** The guidewire is left in place while the dilator is removed. **(d)** Over the guidewire, the catheter is advanced along the tract through the subcutaneous and perirenal tissues. **(e)** The catheter is positioned within the renal collecting system. **(f)** Finally, the catheter’s tip is curled into a pigtail shape by pulling its string using the locking mechanism, completing the procedure

**Fig. 2:**
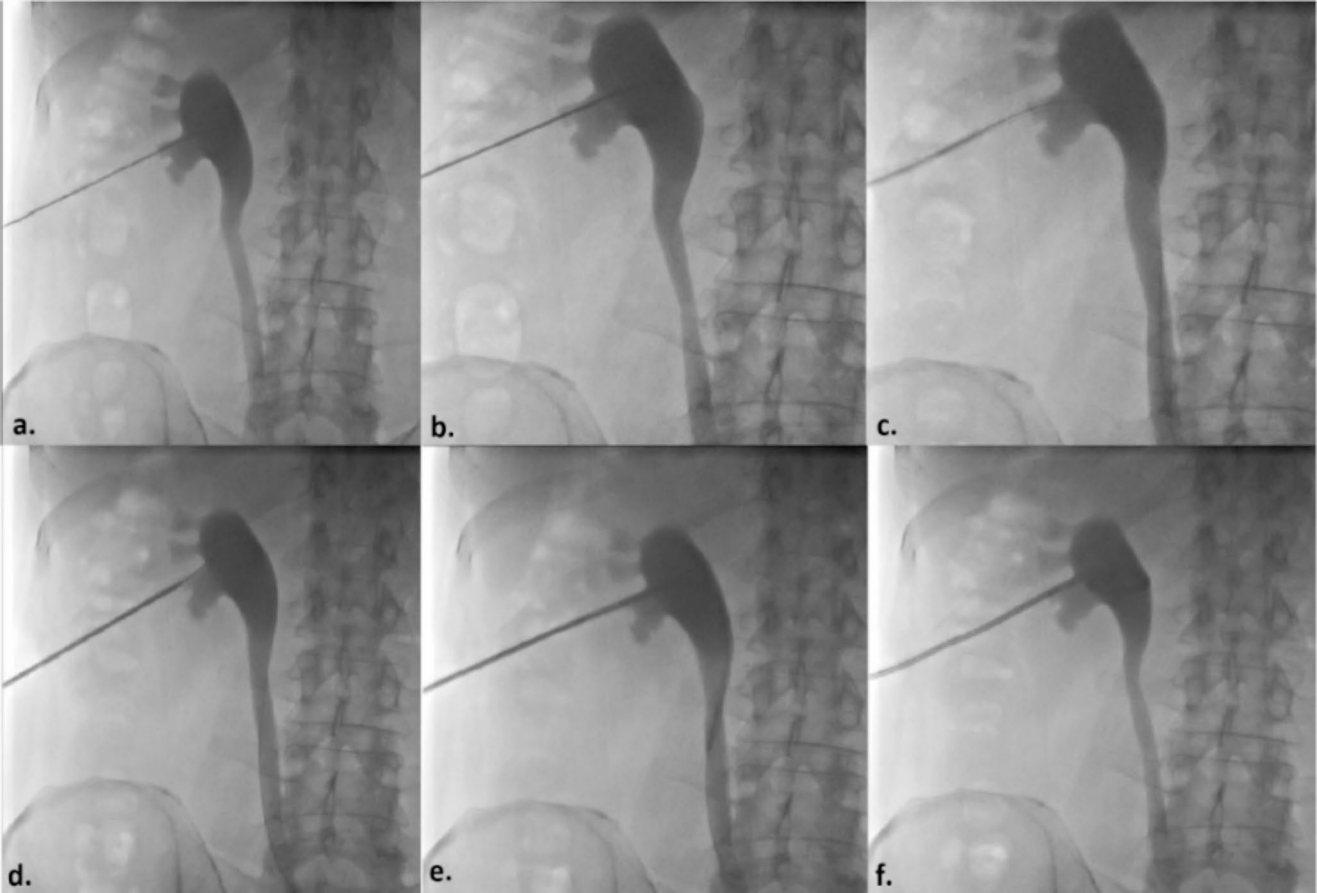
Basic Technique of Percutaneous Nephrostomy Step by Step **(a)** With the patient in the prone position, an 18-Gauge percutaneous needle is inserted into the lower pole calyx of the kidney at a cranial angle via a direct posterior approach. After collecting a urine sample, entry into the collecting system is confirmed with contrast material, followed by antegrade pyelography. **(b)** A 0.035-inch hydrophilic guidewire is advanced through the needle towards the ureteropelvic junction. **(c)** The guidewire is left in place while the needle is removed, and the tract is sequentially dilated to 8 F using a rigid dilator. **(d)** Over the guidewire, the catheter is advanced along the dilated tract through the subcutaneous and perirenal tissues. **(e)** The catheter is then positioned within the renal collecting system. **(f)** Finally, the tip of the catheter is curled into a pigtail shape by pulling its string using the locking mechanism, completing the procedure

**Fig. 3:**
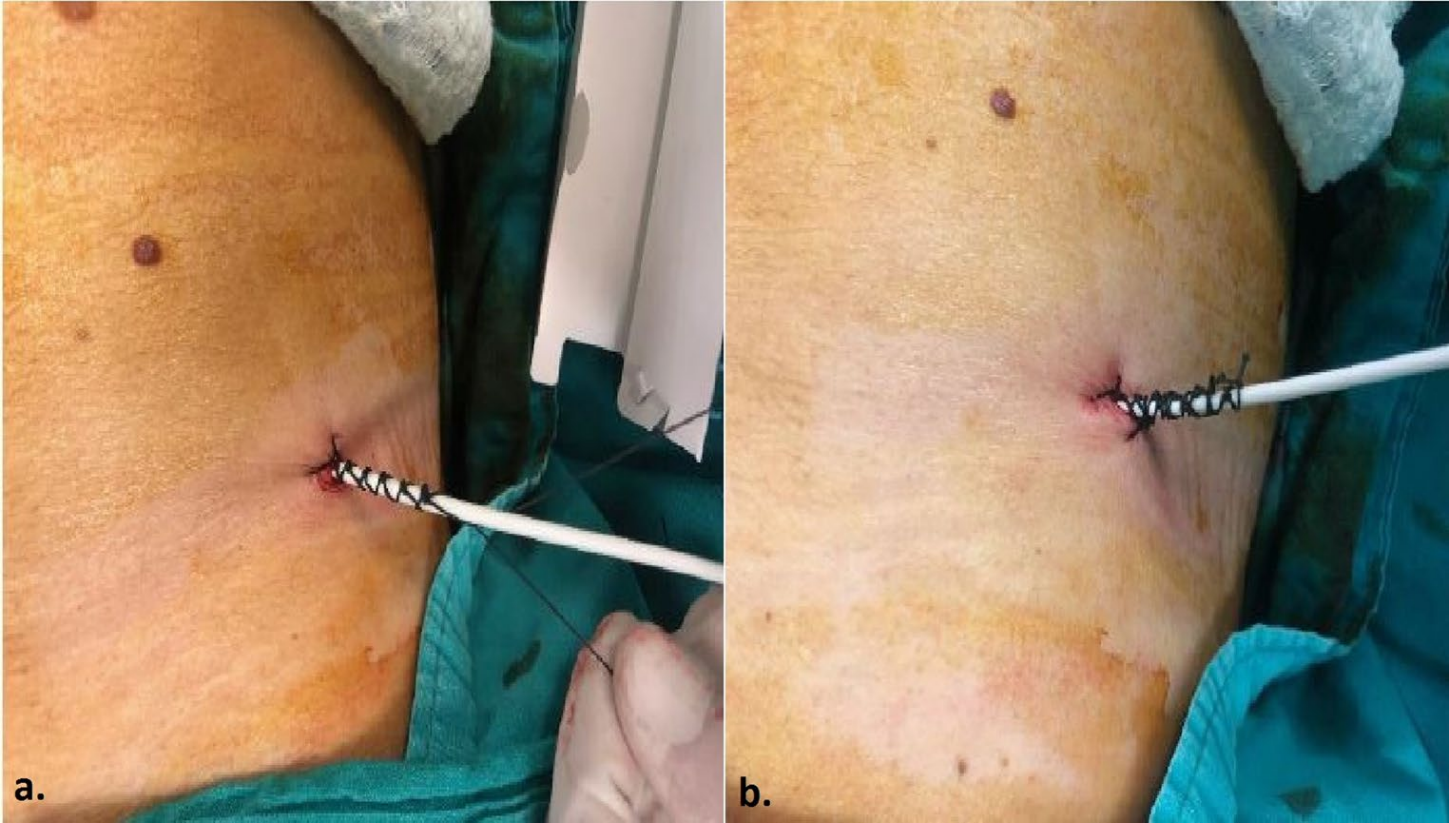
Catheter Fixation in Two Steps (**a, b**): The process involves placing a suture on the skin on each side of the catheter and tying multiple knots over the catheter to form a braid. This is performed first on one side and then repeated on the other

During the procedure, patients were placed in the prone position with two arms and hands gently resting next to the body superiorly. Aseptic technique was carried out and after skin preparation with % 10 povidone iodine, the patients were covered with sterile drapes. Local anesthetic (10–20 mL of lidocaine) was administered subcutaneously and along the planned puncture route, including the perirenal area and existing immatured tract, using a 21-gauge needle. Since PCN catheter insertion and replacement is classified as a clean-contaminated or contaminated procedure, antibiotic prophylaxis with 1 g of ceftriaxone or 1.5–3 g of ampicillin/sulbactam was administered prior to the intervention [2]. In patients whose PCN catheter had been dislodged within the last three days and tract maturation had not yet developed, a new PCN catheter was placed over a 0.035-inch hydrophilic coated guidewire (Terumo Glidewire^®^, Natick, MA, USA). Following the insertion of a 6 French(F) or 8 F dilator at the skin entry point and the administration of contrast material through the established tract under fluoroscopic guidance, a 0.035-inch hydrophilic guidewire was navigated into the collecting system. Once the guidewire’s position within the collecting system was confirmed, an 8 F pigtail tip catheter with pull-string tethered locking system (Flexima, Boston Scientific, Marlborough, MA, USA) was introduced over the guidewire (Fig. 1). For patients with a longer duration since dislodgement, or in cases where access to the renal collecting system could not be achieved using the described method, a new PCN catheter was placed using the standard technique with a fresh puncture. In this technique, the lower pole posterior calyx is accessed via a subcostal approach using an 18-gauge needle under ultrasound guidance. This is followed by the administration of contrast material to confirm access to the renal collecting system, introduction of a 0.035-inch hydrophilic guidewire to the ureteropelvic junction, sequential dilation of the tract with 6 F and 8 F dilators, and finally, the insertion of an 8 F PCN catheter into the renal pelvis (Fig. 2). To prevent inadvertent or spontaneous displacement, the pigtail-shaped tip of the PCN catheters were positioned securely within the renal pelvis and stabilized by fastening the nylon thread tied between the catheter’s opening and the final side hole of the pigtail. Skin fixation was performed using 1/0 natural nonabsorbable silk surgical sutures by the physicians who carried out the procedure, assisted by two nurses with 7 and 11 years of experience in interventional radiology (Fig. 3).

### Abdomen CT measurements and evaluation

All unenhanced abdomen CT examinations were performed with 16-detector row CT scanner (Brilliance; Philips Healthcare, Netherlands). CT images of all patients were evaluated using Osirix software (v12.5.2; Pixmeo SARL, Bernex, Switzerland) on medical imaging workstation by a 4 year radiology resident who completed abdomen imaging training process and a radiologist with 15 year experience on a consensus basis. The readers were blinded to all laboratory, clinical and pathological findings of patients.

**Fig. 4:**
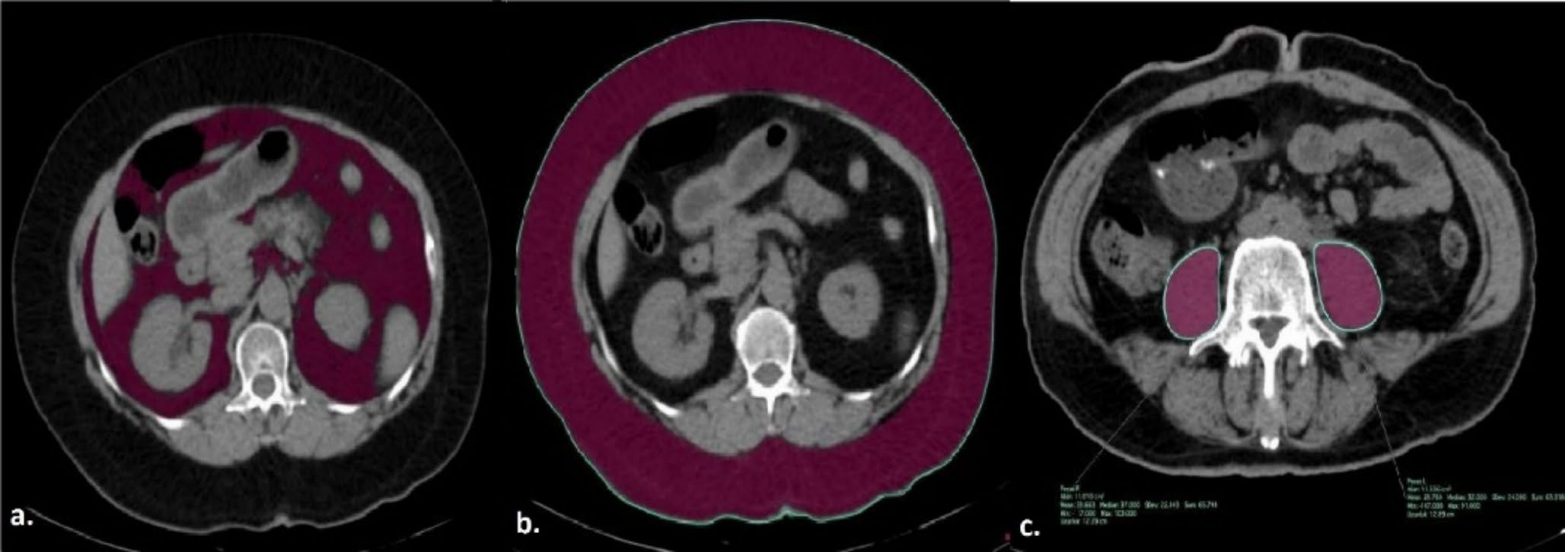
Calculation of intra-abdominal visceral adipose tissue area (**a**), subcutaneous adipose tissue area (**b**), and abdominal perimeter (**b**) at L1 level and the total bilateral psoas muscle area (**c**) at the L4 level

**Fig. 5:**
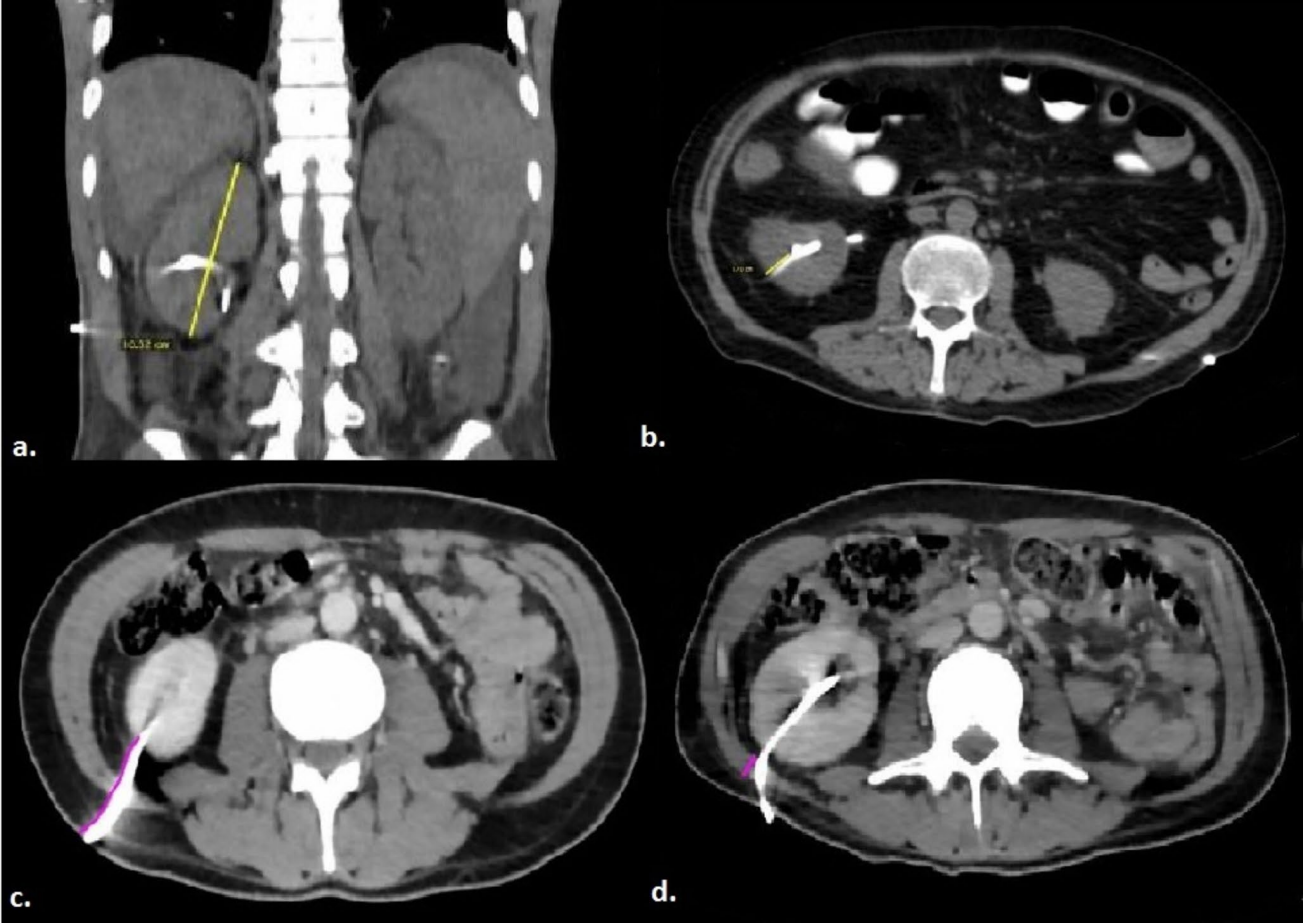
Measurement of renal size on a coronal image (**a**), evaluation of renal parenchymal thickness at the lower pole of the kidney (**b**), measurement of the renal cortex-to-skin distance (**c**), and thickness of the abdominal posterolateral muscle (**d**) on axial images.

The total area of intra-abdominal visceral adipose tissue, subcutaneous adipose tissue, and abdominal perimeter values were calculated by delineating boundaries at the L1 level using the region of interest (ROI) method. Similarly, the total bilateral psoas muscle area was measured at the L4 level using the same technique (Fig. 4). Intra-abdominal visceral and subcutaneous fat tissue measurements on CT were performed using values in the range of -250 to -50 Hounsfield Units (HU), which is considered the fat density range [16]. To normalize these measurements, the obtained values (intra-abdominal visceral adipose tissue area, subcutaneous adipose tissue area and sum of bilateral psoas muscle area respectively) were divided by the square of the patient’s height, and the intra-abdominal visceral adipose tissue index (IAVATI), subcutaneous adipose tissue index (SATI), and psoas muscle index (PMI) were subsequently calculated. Renal size was assessed on coronal images, while the renal cortex-to-skin distance and the total thickness of the posterolateral abdominal muscles (from superficial to deep: external oblique, internal oblique, and transversus abdominis muscles) traversed by the catheter were measured on axial images. Renal parenchymal thickness was evaluated at the lower pole of the kidney on axial images, as the PCN catheter is typically inserted through this region. (Fig. 5)

There is no clear consensus in the literature regarding the optimal location for measuring the areas of intra-abdominal visceral and subcutaneous adipose tissue or the abdominal perimeter on CT. Recent studies assessing intra-abdominal visceral and subcutaneous adipose tissue areas have performed calculations at various levels between L1 and L4, including the first abdominal slice where the lungs are no longer visible and the umbilical level [17–23]. Other publications have measured the abdominal perimeter at levels ranging from T12 to L3, the midpoint between the inferior edge of the 12th rib and the superior margin of the iliac crest, and the first abdominal slice cranial to the iliac crest where the iliac bones are no longer visible [24–27]. Based on these studies, we performed our intra-abdominal visceral and subcutaneous adipose tissue area, and abdominal perimeter measurements at the L1 level.

Recent studies in the literature have highlighted that assessing the total psoas muscle area at all levels between T10 and L5, particularly at the L3/L4 levels, offers a reliable method for the radiological evaluation of sarcopenia [28–31]. In some of our patients, PCN catheters transgressed the abdominal wall muscles at the L3 level, potentially causing artifacts and affecting the accuracy of muscle tissue area and thickness measurements; therefore, based on guidance from studies in the literature, the total psoas muscle area and the thickness of the posterolateral abdominal wall muscles were measured at the L4 level.

ECOG performance status scores are routinely recorded in the medical system for oncology patients, and these scores were retrieved from the medical records. ECOG performance status is a widely used scale to assess a patient’s functional ability, with scores ranging from 0 to 4: 0 indicates fully active, 1 reflects restrictions in strenuous activity but ability to perform light work, 2 indicates limited ability for work but capacity for self-care, 3 reflects significant limitations with self-care and confinement to a chair or bed for more than 50% of waking hours, and 4 represents complete disability and total confinement to bed [32]. However, as bedridden patients were excluded from the study, the analysis included only scores ranging from 0 to 3.

The relationship between inadvertent or spontaneous PCN catheter dislodgement and all aforementioned parameters (IAVATI, SATI, abdominal perimeter, PMI, renal size, renal parenchymal thickness, renal cortex-to-skin distance, and total thickness of posterolateral abdominal wall muscles), as well as the number of spontaneous PCN dislodgements and ECOG scores, were analyzed in detail for the two patient groups.

### Statistical analysis

Statistical analyses were conducted using SPSS version 28.0 (IBM SPSS Statistics for Windows, IBM Corp., Armonk, NY, USA). For parameters related to the relevant side of nephrostomy, such as renal size, renal parenchymal thickness, skin-to-cortex distance, total thickness of posterolateral abdominal wall muscles, and the number of PCN catheter replacements, each nephrostomy replacement procedure was treated as a separate event. Multiple replacements in the same patient (due to scheduled or prophylactic replacements or spontaneous dislodgements) were analyzed independently based on the side involved. In contrast, body fat-muscle composition parameters, including ECOG performance status scores, IAVATI, SATI, and abdominal perimeter, were analyzed on a per-patient basis, with patients categorized as having experienced at least one spontaneous catheter dislodgement or none, irrespective of the side or number of dislodgements.

The normality of the data distribution was assessed using skewness and kurtosis metrics, Kolmogorov–Smirnov, and Shapiro–Wilk tests. Descriptive statistics were expressed as mean, standard deviation, minimum, maximum, and ratio values. The Mann–Whitney U test was used to analyze quantitative independent variables with non-normal distributions, while independent t-tests were applied for normally distributed data. The chi-square test was used to analyze qualitative independent data; however, Fisher’s exact test was applied in cases where the absolute value was less than 5.

Receiver Operating Characteristic (ROC) analysis was performed to determine the optimal cutoff values for the examined variables, utilizing Youden’s Index to optimize sensitivity and specificity. Univariate and multivariate logistic regression analyses were conducted to evaluate the effect sizes of significant factors. A p-value of less than 0.05 was considered statistically significant.

## Results

**Fig. 6:**
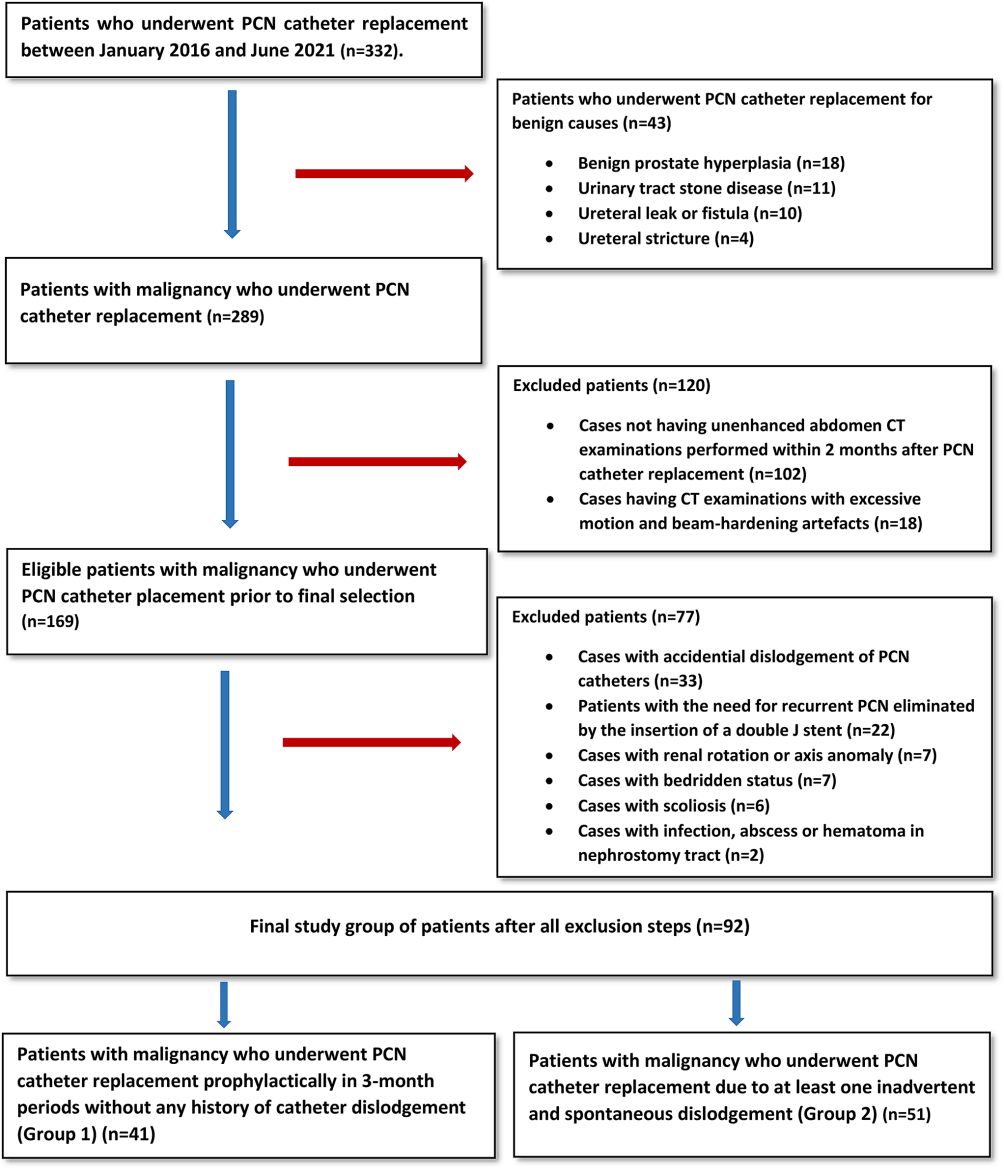
The flowchart of study population

>A total of 92 patients met the inclusion criteria and were included in the study. Group 1 consisted of 41 patients with long-term PCN catheters placed due to malignancy, who had no history of catheter dislodgement, accounting for 84 PCN catheter replacements (right = 38; left = 46). Group 2 included 51 patients with long-term PCN catheters placed due to malignancy, who experienced at least one documented episode of spontaneous catheter dislodgement, with a total of 67 PCN catheter replacements (right = 33; left = 34). In contrast, 240 patients were excluded based on the predefined exclusion criteria. These included 43 patients with benign causes for PCN placement (benign prostatic hyperplasia [*n* = 18], urinary tract stone disease [*n* = 11], ureteral leak or fistula [*n* = 10], and ureteral stricture [*n* = 4]), 102 patients without a computed tomography (CT) scan within two months before or after the nephrostomy procedure, 18 patients with significant imaging artifacts, 33 cases with traumatic or accidental catheter dislodgement, 22 patients whose need for recurrent PCN was eliminated by double J stent insertion via the anterograde approach, 7 cases with renal rotation or axis anomalies, 7 bedridden patients, 6 patients with scoliosis, and 2 cases with infections, abscesses, or hematomas along the nephrostomy tract. The final study selection group process is illustrated in Fig. 6.

For Group 2 patients, the median time to spontaneous PCN catheter dislodgement was 39.2 ± 23.4 days. There was no statistically significant difference in age, sex, height, weight, or body mass index between the patients whose PCN catheters were replaced prophylactically (Group 1) and those with spontaneously dislodged PCN catheters (Group 2) (*p* = 0.162–0.899). Bladder cancer was the most common malignancy in both groups (Group 1: 18 patients, Group 2: 22 patients; 43% of all patients). In Group 1, prostate cancer was the second most common malignancy, affecting 6 patients, while in Group 2, the second most frequent cancers were colonic and rectal carcinomas (Table 1).

**Table 1:** Demographic information and types of malignancies of the study population

		Catheter Dislodgement (-)	Catheter Dislodgement (+)	Total	*P* Value	
		Mean ± Standart deviation	Mean ± Standart deviation	Mean ± Standart deviation		
Age		62 ± 10.5	65.4 ± 12	63.9 ± 11.4	0.162	^t^
Gender	Male	29	34	63	0.677	^X²^
	Female	12	17	29		
Height		1.7 ± 0.1	1.7 ± 0.1	1.7 ± 0.1	0.899	^m^
Weight		68.6 ± 13.7	67.0 ± 13.5	67.7 ± 13.5	0.563	^t^
Body Mass Index		24.9 ± 4.8	24.1 ± 4.1	24.5 ± 4.4	0.409	^t^
Bladder carcinoma		18	22	40	43%	
Colonic carcinoma		5	6	11	12%	
Prostate carcinoma		6	4	10	11%	
Rectal carcinoma		4	6	10	11%	
Cervix carcinoma		3	4	7	8%	
Ovary carcinoma		1	2	3	3%	
Endometrial carcinoma		1	1	2	2%	
Gastric carcinoma		0	2	2	2%	
Lung carcinoma		1	1	2	2%	
Renal cell carcinoma		0	1	1	1%	
Hodgkin lymphoma		0	1	1	1%	
Malign melanoma		0	1	1	1%	
Testicular carcinoma		1	0	1	1%	
Ureteral carcinoma		1	0	1	1%	

t: Independent t-test / m: Mann-whitney u test / X²: Chi-square test(Fischer’s exact test)

There was no statistically significant difference between Group 1 and Group 2 in terms of intra-abdominal visceral adipose tissue area (IAVAT) (*p* = 0.492), intra-abdominal visceral adipose tissue area index (IAVATI) (*p* = 0.412), subcutaneous adipose tissue area (SAT) (*p* = 0.170), subcutaneous adipose tissue area index (SATI) (*p* = 0.210), or abdominal perimeter values (*p* = 0.412). However, a significant difference was found between Group 1 and Group 2 in terms of total bilateral psoas muscle area, psoas muscle index (PMI) and ECOG performance status scores, with p-values of 0.044, 0.04, and 0.0001 respectively (Table 2).

**Table 2:** Comparison of fat-muscle composition parameters, ECOG performance status scores and measurements of tissues traversed by the catheter between patients whose PCN catheters were replaced prophylactically (Group 1) and patients with spontaneously dislodged PCN catheters (Group 2)

Parameter	Catheter Dislodgement (-)	Catheter Dislodgement (+)	*P* Value	
	Mean ± Standart deviation	Mean ± Standart deviation		
PCN Side (Right/Left)	38/46 (45.2%/54.8%)	33/34 (49.3%/50.7%)	0.623	^X²^
Intra-abdominal visceral adipose tissue area (cm²) (IAVAT)	167.5 ± 283.9	109.8 ± 66.8	0.492	^m^
Intra-abdominal visceral adipose tissue area index (cm²/m²) (IAVATI)	63.9 ± 125.7	39.2 ± 22.2	0.412	^m^
Subcutaneous adipose tissue area (cm²) (SAT)	100.1 ± 71.5	83.6 ± 66.8	0.170	^m^
Subcutaneous adipose tissue area index (cm²/m²) (SATI)	37.4 ± 29.5	30.3 ± 23.2	0.210	^m^
Abdominal perimeter (cm)	91.8 ± 10.0	90.0 ± 10.7	0.412	^t^
Total bilateral psoas mucle area (cm²)	17.2 ± 6.6	14.6 ± 5.3	**0.044**	^t^
Psoas muscle index (cm²/m²) (PMI)	6.2 ± 2.2	5.2 ± 1.8	**0.04**	^t^
Eastern Cooperative Oncology Group (ECOG) performance status scores	1.15 ± 0.88	2.08 ± 0.82	**0.0001**	^m^
Renal size (mm)	101.3 ± 14.2	93.9 ± 21.2	**0.025**	^m^
Renal parenchymal thickness (mm)	21.8 ± 3.9	14.4 ± 5.8	**0.0001**	^m^
Renal cortex-to-skin distance (mm)	60.9 ± 18.4	54.9 ± 16.3	**0.039**	^t^
Total thickness of the abdominal posterolateral muscles (mm)	60.9 ± 18.4	8.4 ± 3.0	**0.0001**	^m^
Number of PCN replacements	2 ± 1.2	3.6 ± 2.2	**0.0001**	^m^

t: Independent t-test / m: Mann-whitney u test / X²: Chi-square test(Fischer’s exact test)

In Group 1, 38 patients underwent right-sided replacements and 46 underwent left-sided replacements (total 84), while in Group 2, 33 patients underwent right-sided replacements and 34 underwent left-sided replacements (total 67). There was no statistically significant difference in the side distribution between the two groups (*p* = 0.623). Additionally, in Group 2, renal size, renal parenchymal thickness, renal cortex-to-skin distance, and total thickness of the abdominal posterolateral muscles were significantly lower compared to Group 1 (*p* = 0.0001–0.039), while the number of PCN replacements was significantly higher (*p* = 0.0001) (Table 2).

In the univariate logistic regression analysis, significant effects were observed in distinguishing the two groups based on renal parenchymal thickness, renal cortex-to-skin distance, renal size, and total thickness of the abdominal posterolateral muscles, with p-values ranging from 0.0001 to 0.039. In the multivariate reduced model, significant independent effects were found in separating the two groups based on renal parenchymal thickness and total thickness of the abdominal posterolateral muscles, with p-values of 0.0001 and 0.02, respectively.

Based on the ROC analysis, the area under the curve (AUC) of renal parenchymal thickness in distinguishing patients with and without spontaneous catheter dislodgement was 0.843 (95% Confidence Interval [CI]: 0.769–0.917). When a cutoff value of 16 mm was applied, the sensitivity and specificity were found to be 73.1% and 96.4%, respectively (Fig. 5).

**Fig. 7:**
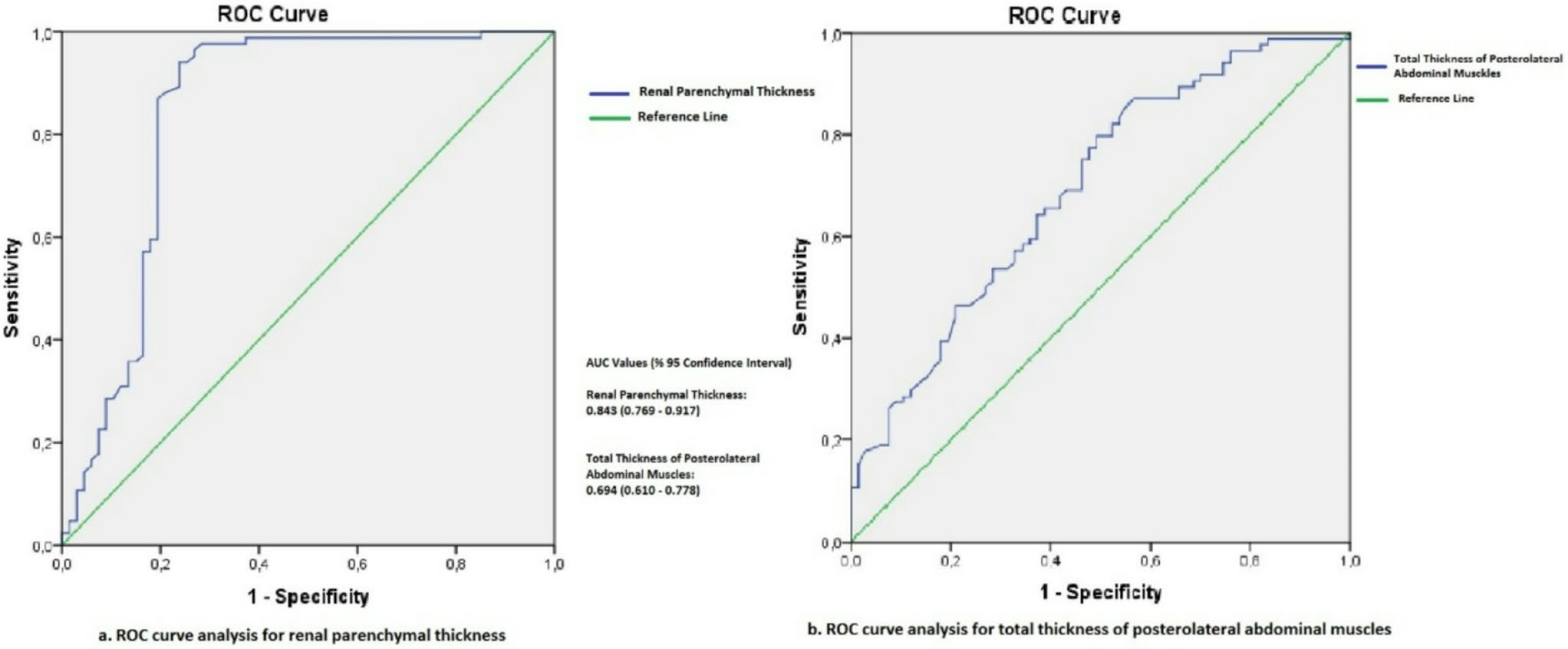
The receiver operating characteristic (ROC) curve illustrates the diagnostic performance of renal parenchymal thickness and the total thickness of the posterolateral abdominal wall muscles in distinguishing between patients with and without spontaneous catheter dislodgement

The ROC analysis revealed an AUC of 0.694 (95% CI: 0.610–0.778) for the total thickness of the posterolateral abdominal wall muscles in differentiating between patients with and without spontaneous catheter dislodgement. Using a cutoff value of 8 mm, the sensitivity and specificity were determined to be 50.7% and 79.8%, respectively (Fig. 7).

## Discussion

In this study investigating the etiology of spontaneous PCN catheter dislodgements in patients undergoing long-term PCN catheterization due to malignancy, our findings revealed no significant association between spontaneous catheter dislodgement and either the abdominal visceral and subcutaneous fat tissue index or abdominal perimeter (*p* = 0.210–0.412). However, significant differences were observed between the two groups in terms of the psoas muscle index and ECOG performance status scores, indicating that reduced muscle mass (sarcopenia) significantly increases the likelihood of catheter dislodgement (*p* = 0.04–0.0001). Furthermore, we observed that the risk of catheter dislodgement was higher in patients with decreased renal parenchymal thickness and reduced total thickness of the posterolateral abdominal wall muscles, as well as in those requiring multiple PCN placements (*p* = 0.0001-0.02). Through ROC analysis, we established cutoff values of 16 mm for renal parenchymal thickness and 8 mm for the thickness of the posterolateral abdominal wall, with respective areas under the curve of 0.843 (95% CI: 0.769–0.917) and 0.694 (95% CI: 0.610–0.778).

Spontaneous dislodgement of PCN catheters, reported in the literature with a wide range of incidence from 1 to 73.5% [7–12], is the most frequently observed complication following percutaneous nephrostomy procedures, while research on its etiology remains limited. This incidence variability is likely attributable to differences in definitions, follow-up durations, and the completeness and consistency of postprocedural monitoring. Additionally, variations in the techniques and extent of external catheter fixation may contribute to the inconsistency in reported rates. Another potential factor is the frequent categorization of this issue under the broad term “catheter dislodgement,” which often lacks clarity on whether the dislodgement is partial or complete. Dislodgement of PCN catheters represents the most frequent reason for catheter dysfunction and must be recognized by physicians performing the procedure, not only due to its relatively high prevalence but also because of the complications and additional interventions it necessitates. Catheter dislodgement increases the risk of infection and bleeding, often necessitating repeated interventional or surgical procedures. According to Meira et al., up to 3.47% of complications require catheter repositioning, contributing to greater workload and financial strain on healthcare systems [33]. Furthermore, the need for urgent reintervention to place a new PCN catheter through the unmatured tract, without creating a new puncture, highlights the urgency and importance of addressing this complication.

The available literature includes limited information, with only a small number of studies exploring the factors contributing to PCN catheter dislodgement. One such study identifies increased body mass index, a history of chemotherapy, diabetes mellitus, low education level, pyelonephritis, catheter-related skin infections, and longer follow-up duration as the main contributors to PCN catheter dislodgement [7]. In a recent study by Panach-Navarrete et al., initial PCN catheter placement by less experienced resident doctors was linked to a higher risk of involuntary displacement. Additionally, pigtail-type PCN catheters were found to dislodge earlier than Foley catheters [9]. In contrast, Bayne et al. reported that the type of nephrostomy tube did not affect dislodgement rates. Instead, they identified body mass index as the primary factor increasing the risk of dislodgement in their study on catheter dislodgements following percutaneous nephrolithotomy [13].

The relationship between body mass index (BMI) and spontaneous PCN catheter dislodgement has been investigated in only a few studies. Bayne et al. reported significantly higher BMI in the dislodgement group (39.7 kg/m²) compared to the non-dislodgement group (30.9 kg/m²), attributing this to catheter fixation to mobile skin associated with fat pannus [13]. Similarly, Alma et al. identified an increased risk of catheter dislodgement with higher BMI (*p* = 0.003) in their malignancy cohort, although their mean BMI (22.0 ± 2.2 kg/m²) was low, consistent with the values observed in our study (24.1 ± 4.1 kg/m² and 24.9 ± 4.8 kg/m² for groups 1 and 2, respectively) [7]. Cachexia is marked by significant loss of fat, muscle, and body weight caused by increased energy expenditure, systemic inflammation, and endocrine dysfunction. It arises from the hypermetabolism of malignant cells, excessive catabolism, inflammation, and reduced food intake due to decreased appetite. This condition likely explains the lower BMI values observed in our study compared to Bayne’s findings. [34]. Unlike these studies, we found no association between BMI and catheter dislodgement risk (*p* = 0.409). Not all patients in our study were in the terminal stage, cachectic, or receiving palliative care. The mean BMI values in both groups (24.1 ± 4.1 kg/m² for group 1 and 24.9 ± 4.8 kg/m² for group 2) were higher than those reported by Alma et al. (22.0 ± 2.2 kg/m²). Despite this, our finding showing no association between catheter dislodgement and BMI warrants further investigation and comparison with a control group without malignancy. Bayne et al.‘s retrospective study evaluated various catheter types inserted through markedly dilated tracts (up to 30 F) after percutaneous nephrolithotomy but provided limited details on catheter sizes, imaging guidance during initial access, and catheter fixation techniques. Unlike their study, which included patients with PCN catheters inserted for a short postoperative period, their conclusions cannot be generalized to oncology patients. While they associated spontaneous catheter dislodgement with increased BMI without detailed subcutaneous fat measurements, we hypothesize that these dislodgements were likely caused by overdilated tracts in their cohort. This highlights the importance of considering tract size and fixation methods rather than solely attributing dislodgements to increased BMI. Furthermore, in the study by Mazon et al., higher rates of local infections, catheter-associated bacteremia, and catheter dislodgement were observed in patients whose central venous catheters were fixed with sutures compared to those fixed with adhesive devices [35]. Although no similar studies exist for PCN catheters, it can be hypothesized that using adhesive fixation devices may reduce the risk of PCN catheter dislodgement, particularly in obese patients with increased subcutaneous fat tissue.

In the study by Navarette et al., conducted mainly on oncology patients, nephrostomy catheters were placed by urologists. Only 8–10 F pigtail catheters were sutured to the skin, while 12 F Foley catheters were left unsecured [9]. They reported that initial nephrostomy placement and procedures performed by inexperienced physicians increased the risk of accidental catheter dislodgement. Although no significant difference in dislodgement rates was found between catheter types, Foley catheters tended to dislodge later than pigtail catheters. Similarly, in the study by Niwa et al., no significant independent risk factors, including catheter type, were identified for predicting nephrostomy catheter displacement. They also found that pigtail catheters had a shorter median time to displacement compared to balloon catheters [12]. In contrast, our study exclusively used 8 F pigtail-tip catheters placed by interventional radiologists with 8 to 15 years of experience. We observed that the risk of spontaneous PCN dislodgement increased with repeated PCN placements, differing from the findings of Navarette et al. We believe this may be related to the use of the same tract for catheter changes, as it likely dilates the pathway and increases susceptibility to catheter dislodgement. This finding aligns with the results reported by Gupta et al. and Saad et al., who observed inadvertent catheter discontinuation rates of 26%, 36%, 53%, and 62% at 6, 12, 24, and 36 months, respectively [10, 36]. However, creating a new tract through a fresh puncture also poses significant risks, such as hemorrhage and injury to adjacent organs. Unlike previous studies, including that of Navarette et al., which did not exclude catheter dislodgements caused by accidental movements, positional changes, or identifiable incidents, our study specifically excluded such cases. Additionally, we focused on a standardized population where all procedures were performed by experienced interventional radiologists using the same catheter type. By consistently suturing the catheters to the skin, we were able to evaluate the direct impact of body composition, kidney dimensions, and surrounding tissue characteristics on catheter dislodgement. As a result, our findings on the etiology of spontaneous PCN catheter dislodgements can improve patient management when procedures are performed correctly, including proper tract dilation, accurate catheter placement, and secure fixation. This will enhance care by providing high-risk patients and their caregivers with better guidance on catheter care, encouraging more frequent follow-ups or shorter replacement intervals. It may also motivate catheter manufacturers to develop designs tailored specifically for these patients, leading to more efficient and effective management.

Renal parenchymal thickness showed notable diagnostic value in predicting spontaneous catheter dislodgement, with an AUC of 0.843 (95% CI: 0.769–0.917), and a cutoff value of 16 mm yielding 73.1% sensitivity and 96.4% specificity. Normal renal parenchymal thickness typically ranges between 15 and 20 mm, and evidence suggests that it decreases with age, as observed in imaging studies [37, 38]. In a study by Kaplon et al., comparing renal parenchymal thickness in patients with obstructive hydronephrosis, the mean thickness was reported as 18.2 mm in the obstructive group and 22.5 mm in the non-obstructive group [39]. If obstruction persists, chronic renal parenchymal atrophy develops, negatively affecting renal function and increasing the risk of spontaneous PCN catheter dislodgement. Therefore, in patients with malignant ureteral obstruction requiring chronic PCN catheter placement, especially those with normal parenchymal thickness and mild or minimal hydronephrosis, timely catheter placement can reduce the risk of spontaneous dislodgement and improve outcomes.

Our study has some limitations. It is a retrospective, single-center study with a limited number of patients, and in our procedures, only a single size (8 F) and type (pigtail) catheter was used. While this limits the generalizability of our results to just one catheter type and size, it also allows for the evaluation of other parameters without being influenced by variations in catheter type or size. Additionally, the inclusion of oncology patients who had an abdominal CT only within 2 months before or after the procedure may have introduced a selection bias. Furthermore, considering the high incidence of sarcopenia and cachexia in patients with malignancies, we believe it would be beneficial to include a control group of patients without malignancies for comparison.

## Conclusion

Spontaneous catheter dislodgement, which can lead to minor or severe complications often requiring urgent intervention, is not an uncommon problem in patients with long-term PCN catheters due to malignancy. It is particularly important for interventional radiologists and urologists performing the procedure to be aware of potential risk factors and management strategies for this clinical issue. In our study of patients with chronic PCN catheters utilizing an 8 F pull-string tethered locking system with a pigtail tip, we identified a significant association between spontaneous catheter dislodgement and both the psoas muscle index and ECOG performance status scores, whereas no significant correlation was observed with abdominal visceral fat tissue, subcutaneous fat tissue volume, or abdominal perimeter. Additionally, we found that catheter dislodgement is more likely with reduced renal parenchymal and posterolateral abdominal wall muscle thickness, as well as with repeated nephrostomy procedures. These findings suggest that reduced thickness in these areas may help identify patients at high risk for spontaneous PCN catheter dislodgement.

## Data Availability

No datasets were generated or analysed during the current study.
